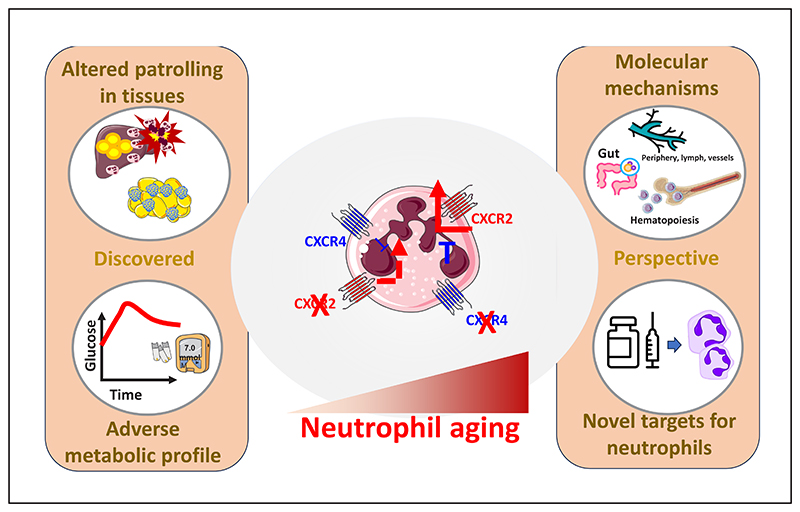# The High Fat Diet Impacts the Plasticity between Fresh and Aged Neutrophils

**DOI:** 10.33696/immunology.5.182

**Published:** 2023

**Authors:** Andrea Baragetti, Giuseppe Danilo Norata

**Affiliations:** 1 Department of Pharmacological and Biomolecular Sciences, Università degli Studi di Milano, Milan, Italy; 2 SISA Centre for the Study of Atherosclerosis, Bassini Hospital, Cinisello Balsamo, Italy

**Keywords:** Neutrophils, Immunometabolism, High Fat Diet

## Abstract

Metabolic alterations induced by unhealthy lifestyles, including obesity and insulin resistance are often associated with increased innate immune response and chronic inflammation. Cholesterol has been identified as a key metabolite driving the activation of the inflammasome and the “epigenetic memory” in long-term living hematopoietic stem cells.

In addition to these mechanisms, the physiological aging of short-living neutrophils is a relevant modifier of their immune competency, as while they egress from medullary niches as “fresh”, fully competent, cells, they turn into “aged”, disarmed cells, when they extravasate into peripheral tissues to fight against pathogens or they reach the spleen for disposal. We recently observed that cardio-metabolic alterations induced by a lipid enriched unhealthy diet critically accelerate this process. Indeed, the chronic feeding with a high fat diet (HFD) results in the increase of aged neutrophils in the circulation and their accumulation in liver. This profile is associated with a deteriorated insulin response and obesity. The HFD primes aged, but not fresh neutrophils, to infiltrate in the liver and promotes inflammation coupled to altered cell immune architecture in visceral adipose tissue. Preventing the aging of neutrophils via selective ablation of CXCR2, reduces the development of obesity and improves the sensitivity to insulin. In humans, plasma levels of CXCL1, one of the cytokines binding CXCR2 and promoting neutrophil aging, are directly associated with abdominal adiposity and fatty liver independently of other risk factors.

Together these findings point to a direct role of aged neutrophils in the development of metabolic disorders.

## Commentary

The intake of high calorie food, which is a risk factor for the development of chronic inflammatory and metabolic conditions and comorbidities, impacts the efficiency of the immune system through different mechanisms that can either constrain or exaggeratedly expands immune cells. Indeed, on one hand when the interferon-gamma signaling of T cells is impaired, such as in obesity [1], the feeding reduces the proresolutive migration of effector lymphocytes in response to infections and in presence of tumours [2], impairs the oral tolerance to antigens by T and B cells infiltrating the intestinal Peyer’s patches) [3] and it increases the risk of mortality in experimental models of pulmonary infection with *P. Aeruginosa* [4]. On the other, the chronic intake of high calorie foods can also promote the acquisition of a hyper-activated of the cell precursors residing in bone marrow (BM), which impacts myeloid cell outputs [5–7]. At the molecular level, this involves an epigenetic reprogramming which promotes the activation of the inflammasome, a complex of cytosolic multiprotein oligomers, that assembles as a consequence of the recognition of either pathogens or inflammatory stimuli [8]. However, an excessive and continuous state of feeding, over-activates the activity of the inflammasome and this results into cellular hyper-proliferation and supra-physiological production of cytokines/chemokines, including interleukin-1β (Il-1β) that, as a result, propagate the inflammatory response over time [7].

Also, cardio-metabolic risk factors trigger an excessive response of the inflammasome, and among them, cholesterol has been identified as a key inducer of the long-term hyper-activation of hematopoietic stem cells (HSCs) residing in the BM. This occurs by establishing a functional epigenetic response known as “trained immunity” [9–11], which affects the activity of a wide set of transcription factors and protein-DNA epigenetic regulators of HSCs physiology [14]. In addition to cholesterol, hyperglycemia also elicits trained immunity of BM-derived macrophages and their precursors, although this occurs via epigenetic activation of the Runt-related transcription factor 1 (Runx1) [12], a crucial transcription factor that regulates the leukemogenic expansion of HSCs. Thus, it is plausible that a sustained intake of refined sugars, which also characterizes the actual dietary patterns [13], can induce inflammatory expansion of hematopoietic progenitor cells via alternative cellular pathways to those triggered by cholesterol. Anyhow, it is to note that triacylglycerols (TGs), rather than cholesterol, represent the main fatty components of foods and, being much more energy-yielding compared to refined sugars, their excessive consumption prominently contributes to the derailment of metabolic homeostasis and to the onset of cardio-metabolic alterations, such as obesity and insulin resistance [14]. Fatty acids (FAs) are released in tissues after the hydrolysis of TGs present in lipoproteins. As such, there is a delicate balance between the delivery of FAs from TG to peripheral cells and their storage in dedicated tissues (for instance, the adipose tissue). In the case of chronic intake of fatty foods, the body initially compensates by increasing the storing capability in specific tissues until a low-grade inflammatory status is generated, which, in turn, triggers the activation of different immune cell subsets via an immunometabolic regulations [15,16]. A role for FAs in the intracellular metabolism of immune cells has been also largely explored. In fact, at cellular level, FAs represent a normal key substrate for the mitochondria to produce energy for different cellular purposes. However, an excess of FAs could drive the overactivation of mitochondria, which results into an increase of oxidative stress and inflammation within the cell [17,18]. Interestingly, these changes in TGs metabolism could be useful when certain immune cells should exert their cytotoxic activity but could be detrimental if it becomes uncontrolled. Also, an excessive substrate of FAs alters the activity of key cellular pathways/components which are essential for the proliferation of the HSCs.

Indeed, long-term living HSCs and the myeloid cell precursors residing in BM (both the common monocytoid progenitors [CMPs], which give rise to monocytes, and the granulocytic progenitor cells [GMPs] which differentiate to neutrophils) largely use oxidative metabolism in the niches [19], but they undergo an immunometabolic reprogramming when the skewing towards the granulocytic lineage is needed to generate short-living neutrophils. Neutrophils, once released from BM predominantly rely on anaerobic metabolism [17,20], despite the availability of oxygen in the blood. Of note, while epigenetic regulations imprint immune competency of neutrophils [5], these cells are programmed to predominantly utilize their DNA as a microbicidal weapon, and generate Neutrophils Extracellular Traps (NETs), to fight against pathogens when they reach target tissues.

It is conceivable to hypothesize that the impact of chronic consumption of calorie-dense, fatty foods on neutrophils goes beyond the activation of the inflammasome and the induction of trained immunity.

Our knowledge on the biology of neutrophils has been also profoundly expanded over the last decade. The classic vision of neutrophils as protagonists of the acute stages of inflammatory responses against pathogens has now been revisited; and these cells have been implicated in rheumatological, autoimmune diseases, atherosclerosis, diabetes and dysmetabolic comorbidities. Moreover, we now recognize a spectrum of neutrophils entities, presenting with different intracellular architecture and with abilities to aggress from BM niches, to distribute among tissues that might differ according to site-specific pathophysiological demands [21–25]. This phenotypic plasticity is simplified with the term “neutrophil aging”, a process that is coordinated by the finely tuned CXCR4/CXCR2 (C-X-C-motif chemokine receptor 4 and 2) axis. CXCR4, by binding the major ligand Stromal cell-derived factor 1 (SDF-1) strengthen the interaction between integrin α4β1 and its counter receptor VCAM-1 in the stromal vascular cells surrounding the niche [26,27], thus anchoring all the hematopoietic cell lineages, including the GMPs and their neutrophilic developmental subsets in BM [21,22]. By contrast, up to eleven chemokines, being produced by macrophages and epithelial cells in response to an inflammatory stimulus, can bind CXCR2, with half maximal effective concentrations (EC50) ranging by 1 nM (Cxcl3 [28]), 3-7 nM (as the case of Cxcl1, Cxcl2, Cxcl6, Cxcl7, Cxcl8 [28,29]), to 10-11 nM (Cxcl5, which is the weakest ligand [28]). Structural difference for some of these chemokines exist between mice and humans and this could translate in different responses in these two organisms. For instance, the murine form of CXCR2 binds to multiple CXCL8-like CXC chemokines, most notably the macrophage inflammatory protein (MIP)-2, which is the murine orthologue of the human 2 and 3 (GRObeta/gamma) [30–32]. The interaction between these chemokines and CXCR2 activates downstream signals that regulate the chemotaxis, the phagocytotic potential and the release of NETs of neutrophils. In addition, Cxcl1/Cxcl2 also coordinate the mobilization from and the homing back to medullary sites by promoting CXCR4 expression [33] and thus establishing a loop able to control the activity of neutrophils during infections [34], where the balance between “fresh” (i.e. over-expressing CXCR4) and “aged” (CXCR2 bright) becomes critical for the survival of the host. All these processes physiologically occur in neutrophils, following a circadian rhythmicity [35] that is also finely dictated by factors that are affected by the environmental and dietary exposure of the host, such as the case of gut microbiota [36]; however, we showed for the first time, that the derailment of the CXCR4/CXCR2 axis also contributes to the metabolic alterations induced by fats-enriched diet [37], thus extending the relevance of neutrophil aging in the context of chronic diseases.

We indeed characterized the development of obesity and metabolic disorders in mice constitutively expressing fresh neutrophils (neutrophil selective CXCR2 deficiency, MRP8Cre+_CXCR2fl/fl) or aged neutrophils (neutrophil selective CXCR4 deficiency, MRP8Cre+_CXCR4fl/fl) after 20 weeks of feeding with a high fat diet (“HFD”). We found that MRP8Cre+_CXCR2fl/fl mice become less obese as compared to MRP8Cre+_CXCR4fl/fl and WT mice and were more responsive to insulin. This was paralleled by reduced liver steatosis. Of note the contrary was observed in MRP8Cre+_CXCR4fl/fl mice, which presented increased lipid accumulation in the liver (Figure 1).

Interestingly neutrophilia, a common feature of MRP8Cre+_CXCR4fl/fl [34], was partly attenuated upon HFD feeding, a finding coupled to an increased accumulation of neutrophils in the liver of these mice but not in other sites of physiological homing such as the spleen, the lung, or the BM [38].

This might suggest that lipid accumulation in the liver could prime neutrophil hepatic infiltration. To explore whether this could be the case under acute conditions, an oral lipid tolerance test (intragastric olive oil gavage) was performed and MRP8Cre+_CXCR4fl/fl mice, but not the other two experimental models, presented a significant accumulation of neutrophils in liver two hours post gavage compared to fasting. Notably, we cannot exclude the relevance of glucose on the activation of neutrophils. Indeed, the expression of CXCR4 in these cells is regulated by the activity of the glucose-6-phosphate transporter [39], a key enzyme for the pentose phosphate pathway. Its genetic ablation in experimental models results into increased CXCR4 expression in these cells, together with intracellular accumulation of glycogen. This observation recapitulates the findings in glycogen storage disease type 1b [39], where neutropenia could be counteracted by treatment with empagliflozin, a glucose lowering drug that inhibits the sodium-glucose co-transporter-2 [40]. Further data to understand the molecular mechanisms beyond these findings are needed.

The different behaviour of aged and fresh neutrophils under this experimental setting is further reflected in their liver proteome. While that of MRP8Cre+_CXCR4fl/fl points to enrichment of proteins related to oxidative metabolism, NETosis and inflammation, the liver proteome of MRP8Cre+_CXCR2fl/fl mice was characterized by the enrichment of pathways related to physiological patrolling and resolutive inflammation.

The translational relevance of these findings was supported by the association that we found between blood neutrophils count and Cxcl1 plasma levels with markers of fatty liver and visceral adiposity in subjects with metabolic syndrome.

These findings highlight that neutrophil aging is both an active player but also a potential target for either lifestyle interventions or therapeutic tools to limit metabolic diseases and particular liver damage. From a nutritional stand point, there is evidence that substituting the intake of refined sugars, or high glycaemic index meals, with vegetables, fibre and healthier food patterns, results into reduction of C-Reactive protein (a common marker of low grade-inflammation) in randomized-controlled interventional trials [41], and with reduced risk of developing clonal haematopoiesis of indetermined potential (CHIP) in leukocytes (a surrogate indicator acquired somatic leukemogenic variations in HSCs) in large epidemiological studies [42]. Likewise, several interventional studies support the anti-inflammatory effect of reducing the dietary intake of saturated fats [14]. Furthermore, increasing the intake of omega-3 fatty acids, particularly eicosapentaenoic acid (EPA) reduces the risk of atherosclerotic events in individuals at elevated CVD risk [43] by stabilizing the cell membrane [44] and by potentially reducing the interaction of neutrophils with endothelial cells, their chemotaxis and transmigration potential [45]. Anyhow, identifying which could be the precise nutritional component that, when a meal is ingested, could elicit the activation of neutrophils is complicated, given the hardwired neuro-hormonal circuits that connect the intestinal epithelium, with the enteric nervous system and the brain. The understanding of such aspects could also be of interest in the context of neuroinflammation.

At pharmacological level, CXCR2-directed therapies are already under investigation for the treatment of cardio-metabolic and liver diseases. Specifically, non-competitive allosteric inhibitors of CXCR2, limit systemic inflammation in streptozotocin-treated mice, reverse diabetes in NOD mice [46], improve hepatic insulin resistance, liver damage and inflammation in experimental models of HFD feeding [47] and in models of non-alcoholic fatty liver disease [48]. Despite these promising findings in experimental models, no beneficial effects of CXCR2 inhibition on residual β-cell function were demonstrated in newly diagnosed patients with autoimmune diabetes [49]. As anti-inflammatory therapies focused at restraining the hyperactivation of the innate arm of the immune system, demonstrated anti-atherosclerotic Volume 5, Issue 5 171 efficacy (e.g., Canakinumab, the antibody against Il-1β [50]) or reached clinical approval or the reduction of cardiovascular risk (as it is the case of Colchicine [51]), identifying strategies to control neutrophil aging without altering their efficacy against infection might help to improve the therapeutic options towards cardioimmunometabolic co-morbidities (Figure 1).

## Figures and Tables

**Figure:**